# New Gratitude Scale for Mexican Youth: Validity and Reliability Analysis

**DOI:** 10.11621/pir.2025.0409

**Published:** 2025-12-15

**Authors:** Jessica N. Acevedo-Ibarra, Esmeralda Contreras-Castañeda, Jorge R. Palacios-Delgado

**Affiliations:** a Valle de México University, Monterrey, Nuevo León, Mexico; b Valle de México University, Santiago de Querétaro, Querétaro, Mexico

**Keywords:** gratitude, positive psychology, scale, validity, youths

## Abstract

**Background.:**

Gratitude is a human strength that promotes well-being; however, it is important to consider culture when constructing scales.

**Objective.:**

To develop and validate a culturally adapted Mexican Gratitude Scale for youth, establishing its psychometric properties: reliability, internal structure, convergent validity, and criterion validity.

**Design.:**

The participants were 442 Mexican young people with a mean age of 20.81 years from three regions of Mexico.

**Results.:**

The initial 28-item scale underwent systematic refinement through Confirmatory Factor Analysis (CFA). This psychometric validation process yielded a final 9-item instrument characterized by a robust two-dimensional factorial structure: personal gratitude and emotional gratitude. The CFA results confirmed this structure through excellent model fit indices (CFI = .98; NFI = .97) and demonstrated significant internal consistency for personal gratitude (ω = .89) and emotional gratitude (ω = .88). The scale provided evidence for convergent validity through significant correlations. Personal gratitude showed moderate associations with optimism (r = .40), positive vision (r = .37), affective resources (r = .30), and hope (r = .43), while emotional gratitude exhibited stronger relationships with optimism (r = .54), positive vision (r = .51), affective resources (r = .42), and hope (r = .52). Discriminant validity was evidenced by young people who do not report any type of mental disease scoring higher on gratitude.

**Conclusion.:**

The psychometric properties of the Mexican Gratitude Scale proved to be acceptable, facilitating its use and application in the general and clinical population for future research.

## Introduction

Positive psychology uses gratitude to give thanks for the good things that have happened (Peterson & Seligman, 2004). Gratitude is a human strength that promotes subjective well-being (Emmons & Crumpler, 2000; Peterson & Seligman, 2004; Watkins, 2004) and contributes to the development of long-term personal resources (Fredrickson, 2004). In addition, it magnifies positive aspects of life and significantly influences personal well-being, interpersonal relationships, and prosocial behavior (Bartlett & DeSteno, 2006; Dunn & Schweitzer, 2005; Langston, 1994; Tsang, 2006; Wood et al., 2010). Likewise, it is associated with adaptive personality characteristics (Emmons & McCullough, 2003; McCullough et al., 2002; Wood et al., 2010), with positive affect, extraversion, forgiveness (Watkins, 2014), satisfaction with life, skills to cope with adverse situations, optimism, motivation, meaning in life, happiness, religiosity, and spirituality (Kerr et al., 2004; McCullough et al., 2002; Seligman & Peterson, 2007; Wood et al., 2008); and it facilitates the development of positive bonds (Bono & Mccullough, 2006). It has also been negatively associated with psychopa-thology in general (Jans-Beken et al., 2019), especially depression, stress, and anxiety symptoms (Althaus et al., 2018; Emmons & Stern, 2013; Seligman et al., 2005; Wood et al., 2008; Wood et al., 2009).

Peterson and Seligman (2004) created a model called VIA Character Strengths and Virtues (CSV), organized by 24 character strengths, or positive traits around six core virtues that characterize an individual and that contribute to their well-being and success in life. Within this framework, gratitude is identiied as one of the key strengths framed by the virtue of transcendence, which fosters meaningful connections with both the universe and others, giving a sense of purpose and meaning to an individual’s life. Peterson and Seligman define gratitude as a feeling of thankfulness and joy in response to receiving a gift, whether a tangible benefit from another person or a moment of peace and bliss evoked by natural beauty. Emmons and Crumpler (2000) argue that it is an emotional state and an attitude toward life, which is a source of human strength to improve personal and relational well-being.

Gratitude has been conceptualized in multiple ways, including as an emotional state, a dispositional trait, and a resilience resource (Emmons & Cumpler, 2000; Fredrickson, 2004; McCullough et al., 2002). McCullough et al. (2002) identify it as an afective trait called grateful disposition or disposition towards gratitude, which is deined as a generalized tendency to recognize and respond with grateful emotion to the benevolence of others in one’s positive experiences and outcomes. McCullough et al. (2004) classify gratitude in three levels: 1) Emotion: a brief, intense response to a positive event; 2) Mood: a relatively stable affective state over time; 3) Affective trait: a generalized tendency to recognize and respond with gratitude.

In recent years, several instruments discussed in this article have been developed to measure gratitude from diferent conceptual perspectives, including as an afec-tive, cognitive, perceptual or expressive process (Tala, 2019), as well as a dispositional trait or affective state (Aparicio et al., 2018; Lambert et al., 2009; Tala, 2019). This diversity of approaches relects the evolution of the concept of gratitude, which has been transformed and expanded over time, demonstrating its complexity and mul-tidimensionality. Among these measures, unifactorial scales include the Gratitude Questionnaire-6 (GQ-6) by McCullough et al. (2002), which focuses on gratitude as an affective trait called grateful disposition or disposition toward gratitude. Th is disposition consists of recognizing and responding with an emotion of recognition to the benefits provided by others in one’s positive experiences and personal achievements. Th e scale consists of six items, although a 5-item version has been suggested for some populations to improve its internal consistency. Th ere is also the GAC Gratitude Checklist (McCullough et al., 2002), which measures states of gratitude over time in a single-factor way, using a list of three adjectives, thus providing a rapid measurement, depending on the time interval of administration.

Additionally, there is the Transpersonal Gratitude Scale by Hlava et al. (2014), a unifactorial scale that evaluates the emotional and spiritual experience of gratitude, with a specific focus on transpersonal gratitude. It consists of 16 items with four dimensions: expression, value of gratitude, transcendent gratitude, and spiritual connection. According to Hlava et al. (2014), gratitude, being a complex emotion, is manifest both in the dynamics of reciprocity generated by received beneits and in deep feelings of emotional bonding.

Multifactorial scales include the Gratitude, Appreciation, and Resentment Test (GRAT) by Watkins et al. (2003), a 44-item scale with three factors: Sense of Abundance, Simple Appreciation, and Appreciation of Others. Th e scale assesses dispo-sitional gratitude as a trait that refers to the predisposition to experience feelings of gratitude. Watkins (2014) suggests that gratitude can be conceptualized as an emotional state, an emotional expression, a character trait or even a virtue.

The Appreciation Scale (AS) by Adler and Fagley (2005) assesses appreciation in multiple dimensions and its relationship to improved well-being. It contains eight subscales: Focus, Awe, Ritual, Present Moment, Personal/Social Comparison, Gratitude, Loss/Adversity, and Interpersonal Appreciation, through 57 items with behavioral and emotional components. Th ese authors highlight that gratitude involves noticing and valuing the meaning of people, events, or objects, generating positive emotions that also strengthen social bonds by expressing them towards others. They also emphasize that, although gratitude may be a dispositional trait, it is a skill that can be learned and cultivated over time, making it a particularly valuable psychological construct.

The 20-item Gratitude Questionnaire measures four components: Interpersonal Gratitude (IG), Gratitude before Suffering (GS), Gift Recognition (GR) and Gratitude Expression (GE). The questionnaire consists of 20 items with cognitive, evaluative, emotional, and behavioral components. A limitation of the results obtained with this questionnaire is that all participants come from a speciic Spanish region (Bernabé-Valero et al., 2014).

Finally, the Gratitude Scale by Alarcón et al. (2014) measures dispositional gratitude through three factors: reciprocity, moral obligation, and sentimental quality. For the authors, gratitude is a positive emotional response from a person (beneiciary) for having received a beneit from another (benefactor), as well as an afective experience that a subject experiences directly, allowing them to immediately grasp the positive qualities of a human action. This scale, composed of 18 items, integrates emotional and behavioral components, and to date has been applied exclusively to the Peruvian adult population.

Despite advances in gratitude measurement, it is important to consider culture when constructing scales, because it is essential for understanding social behavior and personality (Díaz-Loving, 2020; Palacios, 2015; Triandis & Suh, 2002). Scales such as the GQ-6 (McCullough et al., 2002) and the GRAT (Watkins et al., 2003) focus predominantly on emotional or dispositional aspects. Th erefore, it would be important to consider appreciation in contexts of adversity and interpersonal factors (Bernabé-Valero et al., 2014).

Additionally, most of these instruments have been validated in Anglo-American and European populations, which restricts their applicability in diverse cultural and demographic contexts (Alarcón, 2014; Bernabé-Valero et al., 2014). For example, Ruby et al. (2012) identified significant differences in emotional preferences between collectivist cultures, highlighting that, for Mexicans, the open expression of emotions is fundamental. In line with these indings, Corona et al. (2020) reported that Latino Americans show willingness to experience and express gratitude more frequently and intensely than East Asian Americans, considering it more appropriate and desirable. These findings demonstrate cultural differences in the experience and valuation of positive emotions, underscoring the influence of the cultural context on emotional experiences. Furthermore, although the GQ-6 has been validated in the Mexican population (Quezada, 2023), it focuses on the more emotional aspects of gratitude (Wood et al., 2008). Additionally, Anderson (2005) points out that, although McCullough et al. (2002) incorporate in their definition of gratitude the idea that it is a response to the contribution of others to personal well-being, they do not establish limitations on the items, which allows some to allude more to what is understood by appreciation than to gratitude itself and highlights that none of these items clearly explains the contribution of a specific agent.

Although some authors have highlighted the importance of integrating impersonal forces and interpersonal agents in measuring gratitude (Anderson, 2005; Wood et al., 2010), few instruments address this approach comprehensively. Th erefore, we consider it important to understand gratitude as a multidimensional construct that includes not only the interaction between people, but also the recognition and appreciation of life that leads humans to a state of tranquility and happiness. For this reason, the creation of a new scale of gratitude as a feeling and source of human strength that fosters tranquility and happiness, culturally adapted, becomes a necessity to advance psychological research and practice (Díaz-Loving, 2020; Palacios, 2021). Th is scale would make it possible to address the sociocultural needs of the Mexican population, identify strengths in young people, and contribute to the development of evidence-based psychosocial interventions. Such interventions could promote psychological well-being and mental health, as a complement to psychotherapy to improve outcomes (Wong et al., 2018).

The objectives of our study are to design and validate a new scale to measure gratitude in a sample of young Mexicans, as well as to obtain convergent validity through the relationship with optimism and criterion validity considering the presence or absence of a neurological or psychiatric disease. Our hypotheses are the following: 1) the gratitude scale will obtain a better it to the data when considered as multifacto-rial compared to a single-factor scale; 2) our gratitude scale will have a strong, positive, and significant association with optimism; 3) gratitude scores measured by our scale will be statistically higher among young people who do not report any type of neurological or psychiatric disease.

## Methods

### Participants

The participants were selected through a non-probabilistic method that included 442 young people; 67.6% were women, 30.5% were men, and 1.8% preferred not to specify their gender. The mean age was 20.8 years (SD *=* 5.7). The majority of participants were single (92.1%), while 3.4% were married, 3.6% were in a common law union, .5% were divorced and .5% were widowed. Of the young people included in the study, 68.3% were students, and 31.7% were both studying and working. Th e participants were grouped into three regions of the country according to their place of residence: 41.2% were from the central region, 31.2% from the northern region, and 27.6% from the southern region.

### Questionnaires

*Mexican Gratitude Scale (MGS)*. The MGS is a 28-item Likert-type instrument with response options ranging from 1 (no days of the week) to 5 (almost every day of the week), where higher scores indicate greater gratitude. In this study, gratitude is conceptualized as a feeling of thankfulness expressed toward others, serving as a psychological resource that fosters appreciation for life, personal experiences, and interpersonal connections, ultimately promoting tranquility and happiness. A specific instrument was developed for this study from the theoretical perspective of the model called VIA Character Strengths and Virtues (CSV) (Peterson & Seligman, 2004).

*Mexican Optimism Scale (MOS)*. The MOS comprises 15 items measuring optimism as an integrated emotional repertoire of personal resources for navigating life circumstances positively. It includes three subscales: affective resources, positive vision, and hope, rated on a 5-point Likert scale (Palacios-Delgado & Acevedo-Ibarra, 2023). Th e MOS demonstrates strong construct validity (CFI = .96, NFI = .95, TLI = .96, RMSEA = .05) and reliability (ω = .83 for affective resources, .88 for positive vision, and .88 for hope).

### Procedure

Data were collected from September 2023 to December 2024. Young university students were contacted and invited to participate in the research. After receiving an explanation of the study’s purpose, participants who agreed to take part signed an informed consent form and completed the digital instruments immediately via Google Forms. Th e form included sociodemographic and clinical questions, along with psychological scales, and participants were encouraged to respond truthfully. The research was approved by the Health Sciences Investigation Committee of the Universidad del Valle de México on August 25, 2023, with the registration number PFUVM2023-003.

Statistical analyses were conducted using the JASP Program version 0.9.2. First, distribution and item discrimination were reviewed. Univariate and multi-variate normality were assessed for the total sample, applying the Shapiro-Wilk test along with skewness and kurtosis tests. Psychometric data such as item-total correlation criteria was used, removing those items that obtained a correlation lower than .40. The results indicated the suitability of the items. To continue with the analysis, Conirmatory Factor Analysis (CFA), was applied through the maximum likelihood estimation approach. To evaluate goodness of fit, we computed the chi-square (χ^2^), root-mean-squared error of approximation (RMSEA), comparative fit index (CFI), relative fit index (RFI), incremental fit index (IFI), normed fit index (NFI), and standardized root mean residual (SRMR). To evaluate the cut-off and determine model fit, we followed the guidelines published by the *European Journal of Psychological Assessment* (Schweizer, 2010). The internal consistency of the resulting factors in the scale was assessed using Cronbach’s Alpha and McDonald’s Omega.

To produce further information about the construct validity of the scale, discriminant validity analysis (including average variance extracted: AVE) and the HTMT ratio were performed. For AVE, values > .50 are required, while for HTMT, values < .90 are acceptable (Henseler et al., 2015). To determine convergent validity, Spearman’s correlations between the scores obtained for gratitude and those obtained for the optimism total scale were carried out. Mann-Whitney U tests were used to determine criterion validity, with a signiicance level of p < .05. Efect sizes were reported as *r* **[r = z/Vñ]** for non-parametric data and interpreted using the conventional metrics as small = .10, medium = .30 and large = .50 (Fritz et al., 2012).

### Results

*Table 1* presents descriptive statistics of the individual items of the scale. For skewness and kurtosis, many items fell within the ± 1.5 range (Ferrando & Anguiano-Carrasco, 2010), while others had higher values. Nonetheless, Shapiro-Wilk’s normality tests showed that all items were non-normally distributed (p < .01).

**Table 1:** Descriptive Statistics for Each Item

Item	*M*	*SD*	*Gl*	*g2*	*SW*	*p*
1. I feel grateful for things. (Me siento agradecido (a) con las cosas.)	3.9	1.0	–.8	–.2	.8	< .001
2. I am grateful to those around me, such as family and friends. (Soy agradecido con quién me rodea, como familia y amigos.)	4.2	.9	–1.2	.7	.7	< .001
3.I feel and express gratitude towards something or someone. (Siento y expreso agradecimiento hacia algo o alguien.)	3.9	1.1	–.8	–.2	.8	< .001
4. I am grateful and satisfied with my achievements and successes. (Me encuentro agradecido y satisfecho con los logros y éxitos que tengo.)	3.6	1.2	–.4	–.9	.8	<.001
5. I am grateful for what I have. (Soy agradecido con lo que tengo.)	4.2	.9	–1.1	.3	.7	<.001
6. I feel grateful for who I am. (Me siento agradecido con quién soy.)	3.8	1.1	–.6	–.7	.8	<.001
7. I feel grateful to someone who helped me. (Me siento agradecido (a) hacia una persona que me ayudó.)	4.3	.9	–1.2	.8	.7	<.001
8. I feel grateful to those who are there for me. (Me siento agradecido con las personas que están para mí.)	4.4	.8	–1.5	1.5	.6	<.001
9. I let other people know how much I value them. (Le hago saber a las demás personas lo mucho que valen.)	3.8	1.1	–.7	–.4	.8	< .001
10. I am grateful for an act of help or kindness towards me. (Agradezco un acto de ayuda o amabilidad hacia mi persona.)	4.2	1.0	–1.3	.7	.7	<.001
11. I acted in a grateful manner. (Actuó de una forma agradecida.)	4.2	.9	–1.1	.6	.7	<.001
12. I am grateful to someone for their good actions. (Soy agradecida (o) con alguien por sus buenos actos.)	4.3	.9	–1.3	1.2	.7	<.001
13. I am grateful to a person for the actions they have performed for me, without expecting anything in return. (Estoy agradecido hacia una persona por los actos que ha realizado hacia mí, sin esperar nada a cambio.)	4.3	.8	–1.3	1.1	.7	<.001
14. I appreciate the people I’m with. (Aprecio a las personas con quién estoy.)	4.4	.8	–1.2	.7	.7	<.001
15. I feel esteem for someone who has done me a favor. (Siento estima por alguien que me haya hecho algún favor.)	4.2	.9	–1.1	.6	.7	< .001
16. I am kind to people who have helped me at some point. (Soy gentil con las personas que me han ayudado en algún momento.)	4.4	.8	–1.4	1.5	.7	< .001
17. I feel peace of mind for what I have. (Siento tranquilidad por lo que tengo.)	3.9	1.0	–.8	–.2	.8	< .001
18. I feel satisfaction for what I have. (Siento satisfacción por lo que tengo.)	3.9	1.0	–.7	–.4	.8	< .001
19. I feel happiness for what I have. (Siento felicidad por lo que tengo.)	4.1	1.0	–.9	–.1	.7	< .001
20. I show appreciation to someone who once helped me. (Muestro aprecio hacia la persona que alguna vez me ayudó.)	4.3	.8	–1.2	.7	.7	< .001
21. I appreciate what I have in life. (Aprecio lo que tengo en la vida.)	4.2	1.0	–1.0	.0	.7	< .001
22. I appreciate the good actions other people do for me. (Aprecio los buenos actos que otras personas hacen por mí.)	4.4	.8	–1.4	1.3	.6	< .001
23. I can appreciate things. (Puedo apreciar las cosas.)	4.2	.9	–1.1	.2	.7	< .001
24. I appreciate the moment I’m in. (Aprecio el momento en el que vivo.)	4.0	1.0	–.9	–.0	.8	< .001
25. I acknowledge someone who has done me a favor or provided a service. (Reconozco la persona que ha hecho un favor o prestado un servicio.)	4.3	.9	–1.2	.5	.7	< .001
26. I feel sentimental value for what I have. (Le doy un valor sentimental a lo que tengo.)	4.2	.9	–1.0	–.0	.7	< .001
27. I recognize other people’s loyalty to me. (Reconozco la lealtad de las (los) demás personas hacia mí.)	4.3	.9	–1.3	.9	.7	< .001
28. I appreciate what I have, comparing it with other situations where it could be worse. (Valoro lo que poseo, comparando con otras situaciones donde pudiera estar peor.)	4.1	1.0	–1.0	.3	.7	< .001

*Note. M = Mean, SD = Standard Deviation, gl = Skewness, g2 = Kurtosis, SW = Shapiro-Wilk*

The factorial structure proposed with a CFA model on the total sample was tested. Th e estimation method used was maximum likelihood. Two models were evaluated: the first was unifactorial and the second was bifactorial, considering the modiication indices to ind the appropriate it on the scale. Adjustments were made by eliminating the elements that did not conform to the established model. First, the item-total correlation was examined, discarding those items with < .40 values. Next, a one-dimensional CFA was conducted, which showed a poor it to the measurement model. Based on the modiication indices, redundant items or those with high residual covariance were removed, resulting in the elimination of 19 items (*Table 2*).

**Table 2:** Factor Structure and Items Loadings of Gratitude Scale

					95% Confidence Interval	
Factor	Indicator	Estimate	*z*-value	*P*	Lower	Upper
Personal gratitude	Item 12	.7	21.2	< .001	.7	.8
	Item 11	.6	18.3	< .001	.6	.7
	Item 8	.6	18.6	< .001	.5	.7
	Item 20	.6	18.6	< .001	.5	.7
	Item 14	.6	19.4	< .001	.5	.7
Emotional gratitude	Item 19	.9	23.4	< .001	.8	1.0
	Item 17	.8	2.8	< .001	.8	.9
	Item 24	.8	19.1	< .001	.7	.9
	Item 6	.8	17.0	< .001	.7	.9

**Figure 1.**
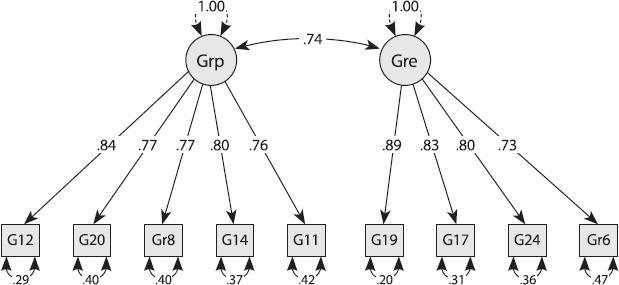
Confirmatory analysis of Mexican Gratitude Scale

*Table 3* shows that absolute goodness of fit, incremental adjustment, and parsimony adjustment indices were adequate (Hu & Bentler, 1999). The scale adequately explained gratitude, with coefficients ranging from 0.65 to 0.92 (*Figure 1*). The AVE value exceeded the recommended minimum, .62 for the personal factor and .63 for emotional factor. The HTMT ratio was .75, which meets discriminant validity criteria.

**Table 3:** Fit Measures of the Models Tested

CFA Model	χ^2^	X^2^/df	CFI	TLI	NFI	RFI	IFI	RMSEA
One Factor	1453.33	5.76	.85	.83	.82	.80	.85	.10
Two factors	75.23	2.89	.98	.98	.97	.96	.98	.06

The reliability by internal consistency Alpha and Omega was calculated. The results revealed good values for personal gratitude (α = .89 [IC95% = .87 - .90] ; ω = .89 [IC95% = .87 - .90]), and emotional gratitude (α = .88 [IC95% = .86 - .92]; ω = .88 [IC95% = .86 - .92]).

To assess convergent validity, correlations between the gratitude scale and the optimism scale and its subscales were analyzed. The Mexican Gratitude Scale demonstrated convergent validity, with positive Spearman’s correlation coefficients with the hypothesized related construct. The two factors personal and emotional gratitude and total score were correlated with the optimism construct (*Table 4*). A strong relationship was observed between emotional gratitude, positive vision, and hope.

**Table 4:** Descriptive Statistics and Correlations of Gratitude Scale and Optimism

Factor	Optimism	Positive vision	Affective resources	Hope	*M*	*SD*
Personal gratitude	40***	.37***	.30***	.43***	21.8	3.6
Emotional gratitude	.54***	.51***	.42***	.52***	15.7	3.7

Note. *p < .05, **p < .01, ***p < .001, M = Mean, SD = Standard Deviation. The reported mean refers to the mean of the sum of the items that make up each factor.

To assess criterion validity, each gratitude factor was compared between participants with and without reported disease (presence n = 46; absence n = 396). The data showed statistically signiicant diference in both gratitude factors and total score, with higher scores among young people who do not report any type of mental disease: personal (presence: M = 2.1, SD = 4.5; absence: M = 22.0, SD = 3.4; U = 677.0; p < .01; r_B_ = .2), emotional (presence: M = 14.2, SD = 3.8; absence: M = 15.9, SD = 3.7; U = 6661.0; p < .01; r_B_ = .2) and total score (presence: M = 34.4, SD = 7.6; absence: M = 37.9, SD = 6.5; U = 6474.5; p < .01; r_B_ = .2).

## Discussion

The study aimed to design a new gratitude scale for young Mexicans, assessing internal structure validity, convergent validity (with optimism), discriminant validity (AVE and HTMT), and criterion validity (association with neurological or psychiatric disease). The initial 28-item instrument was refined to a 9-item scale, demonstrating validity, reliability, and cultural appropriateness.

Gratitude was deined as a feeling of gratefulness or thankfulness expressed to others, serving as a source of strength that facilitates recognizing and appreciating life, one’s own experiences, and others, leading to a state of tranquility and happiness. It was operationalized using 28 items, resulting in a inal 9-item scale that, according to CFA, confirms a two-factor structure: personal gratitude and emotional gratitude.

Personal gratitude contains five items, which are found in *Table 2*. Th ese items are consistent with theoretical assumptions that consider gratitude toward a speciic person for the beneit they have provided or simply for their existence; gratitude as a source of strength refers to noticing and being grateful for the good things that happen, as well as taking the time to express gratitude (Peterson & Seligman, 2004). Likewise, gratitude is a sense of thankfulness and appreciation for life that can be expressed toward others, as well as toward impersonal sources such as nature (Emmons & Shelton, 2002). Emotional gratitude consists of four items, which are found in *Table 2*, and are addressed by a study that indicates that gratitude is an emotional state and an attitude toward life, serving as a source of human strength to improve personal and relational well-being (Emmons & Crumpler, 2000).

The convergent validity results obtained through the correlation with optimism show that personal gratitude and emotional gratitude are positively associated with optimism, as well as with its factors including positive vision, afective resources and hope. These results are consistent with other studies that show these associations (Almansa et al., 2024; Bazargan-Hejazi et al., 2023; Biber et al., 2020; Emmons & Mc-Cullough, 2003; Hahn et al., 2024; Puente-Díaz & Cavazos-Arroyo, 2022). Th ese results may be due to the fact that gratitude and optimism represent positive emotional states. According to Fredrickson’s theory (1998), positive emotions expand an individual’s momentary repertoire of thought-action, which in turn can strengthen their lasting personal resources. In other words, cultivating positive emotions not only counteracts negative emotions, but also expands people’s habitual ways of thinking and strengthens their personal resources to cope with stress and be more resilient in the face of adverse situations. This is similar to what the results of other studies suggest (Acevedo-Ibarra et al., 2019; Acevedo-Ibarra et al., 2020; Acevedo-Ibarra et al., 2022; Palacios-Delgado et al., 2024).

Regarding optimism, it is possible that afective states, positive emotional resources, and a positive perspective on life and adverse situations (Palacios-Delgado & Acevedo-Ibarra, 2023) lead people to feel grateful, which is expressed to others. Meanwhile, hope as the connection with oneself or with others, which is linked to something external such as family and spirituality to face adversity, and the adaptation of negative changes to positive changes in life (Palacios-Delgado & Acevedo-Ibarra, 2023), is what enables the person to experience gratitude facilitating their ability to recognize and appreciate life, their experiences and others, and therefore achieve a state of tranquility and happiness. Therefore, it is possible that in our culture young people use emotional gratitude, optimism, and hope to face demanding situations, leading them to a state of tranquility and happiness, which would explain the strong association between the variables.

In relation to criterion validity, the results show that young people who do not report mental or neurological disease have higher gratitude scores. Our results are consistent with other research (Hao et al., 2024; Liang & Xiang, 2024; Lin, 2015; Liu et al., 2022) where gratitude is observed to be significantly and negatively associated with depression and anxiety. Likewise, the positive effects of gratitude on depression in hospitalized psychiatric patients are highlighted (Kim & Jun, 2021). Furthermore, gratitude has been observed to foster social support and hope, protecting people from stress and depression (Feng & Yin, 2021).

Regarding the analysis of internal consistency, the Cronbach’s Alpha and McDonald’s Omega coefficients obtained indicate an acceptable level of reliability. Our results were in line with these estimates in scales from diferent cultural contexts reported by other researchers (Alarcón, 2014; Bernabé-Valero et al., 2014; McCullough et al., 2002; Watkins et al., 2003) as well as in the Mexican context (Quezada, 2023), indicating that the reliability is adequate for the use of the scale.

One of the main implications of good reliability is the ease of detecting significant changes in levels of gratitude over time or in response to psychological interventions (Cunha et al., 2019). Adequate reliability also impacts the sensitivity to change of the scale and its ability to detect clinically important changes in the measured construct (Palacios-Delgado & Acevedo-Ibarra, 2023). An unreliable scale may not be sensitive enough to capture subtle but signiicant changes in gratitude (Rash et al., 2011), which could have direct practical consequences for assessing the effectiveness of interventions (Palacios-Delgado et al., 2025).

In addition, the new Mexican Gratitude Scale is culturally adapted, and captures personal and emotional aspects of young Mexicans, framing gratitude as a feeling that provides human strength and that contributes to personal and relational well-being (Emmons & Crumpler, 2000; Peterson & Seligman, 2004). Thus, the scale can be used in clinical and non-clinical samples. Furthermore, our results support socioculturally tailored psychosocial interventions that promote personal and emotional gratitude, while also incorporating optimism, particularly positive vision and hope, to improve mental health in young people (Palacios-Delgado et al., 2024). Another study shows that, in situations of high adversity, the development of optimism associated with hope and gratitude will have greater protective effects on people’s well-being and will also be more effective in reducing students’ anxiety (Almansa et al., 2024).

Accordingly, we agree with Bazargan-Hejazi et al. (2023) regarding the development of culturally adapted strategies that foster optimism, hope, and gratitude in minority groups, which promote development and well-being. This is because interventions that cultivate positive emotions are particularly suitable for preventing and treating problems such as anxiety, depression, aggression, and stress-related health problems (Fredrickson, 2000). We also agree with Feng and Yin (2021), who suggest that psychological interventions should not only focus on alleviating negative psychological problems, but also on their positive psychological development. Additionally, gratitude and optimism predict greater psychological well-being and happiness in Mexicans (Martell-Muñoz et al., 2025). Therefore, in future studies we suggest testing clinical interventions targeting gratitude (Seligman et al., 2005; Wood et al., 2010) and optimism such as positive vision, affective resources, and hope (Palacios et al., 2024) to improve young people’s mental health, happiness, and biopsychosocial well-being.

## Conclusion

The results demonstrate a new two-dimensional gratitude scale was obtained in young Mexicans, which conirms its validity, reliability, and cultural adaptation. The final scale consists of nine items measuring two factors: personal gratitude and emotional gratitude. Convergent validity established expected correlations with optimism, positive vision, affective resources, and hope. Criterion validity revealed statistically significant differences according to mental or neurological disease. Th ese psychometric properties indicate that the new gratitude scale can assess the construct in both nonclinical and clinical samples, as well as evaluate gratitude-based interventions in future research.

## Limitations

The study has some limitations. The sample was non-probabilistic, which could limit the generalizability of the results, and thus the results should be viewed with caution. Probabilistic methods, larger samples, and studies with other population groups, such as adults, older adults, and those with chronic illness, are needed to conirm the psychometric properties of our scale. Another limitation is that measurements of negative emotional states were not included to assess discriminant validity, so future studies should test these associations with variables such as depression and anxiety. One methodological limitation relates to the estimator, since for the CFA we used maximum likelihood, assuming continuity and normality in ordinal data, which may affect the accuracy of the estimates. Therefore, the interpretation of the results should be approached with caution.

Our results ofer important contributions. First, our scale considers gratitude as a human strength that enables the recognition and appreciation of life, not just interpersonal interactions. It is a scale that prioritizes emotional aspects and is adapted to the sociocultural characteristics of our population, making it suitable for use in our context and potentially in Latin America. Moreover, we have established criterion validity based on the presence of psychiatric or neurological disease, which supports the use of gratitude in clinical interventions consistent with studies suggesting that cultivating gratitude in university students may prevent depression (Liu et al., 2022). Furthermore, gratitude can enhance positive psychosocial factors such as optimism and hope, decreasing anxiety (Almansa et al., 2024).
